# The link between obesity and insulin resistance among children: Effects of key metabolites

**DOI:** 10.1111/1753-0407.13460

**Published:** 2023-08-25

**Authors:** Wu Yan, Su Wu, Qianqi Liu, Qingqing Zheng, Wei Gu, Xiaonan Li

**Affiliations:** ^1^ Department of Children Health Care Children's Hospital of Nanjing Medical University Nanjing China; ^2^ Department of Endocrinology Children's Hospital of Nanjing Medical University Nanjing China; ^3^ Institute of Pediatric Research, Nanjing Medical University Nanjing China

**Keywords:** children, insulin resistance, metabolites, obesity, 肥胖症, 胰岛素抵抗, 代谢物, 儿童

## Abstract

**Background:**

Childhood obesity became a severe public health challenge, and insulin resistance (IR) was one of the common complications. Both obesity and IR were considered as the basis of metabolic disorders. However, it is unclear which common key metabolites are associated with childhood obesity and IR.

**Methods:**

The children were divided into normal weight and overweight/obese groups. Fasting blood glucose and fasting insulin were measured, and homeostasis model assessment of insulin resistance was calculated. Liquid chromatography–tandem mass spectrometry was applied for metabonomic analysis. Multiple linear regression analysis and correlation analysis explored the relationships between obesity, IR, and metabolites. Random forests were used to rank the importance of differential metabolites, and relative operating characteristic curves were used for prediction.

**Results:**

A total of 88 normal‐weight children and 171 obese/overweight children participated in the study. There was a significant difference between the two groups in 30 metabolites. Childhood obesity was significantly associated with 10 amino acid metabolites and 20 fatty acid metabolites. There were 12 metabolites significantly correlated with IR. The ranking of metabolites in random forest showed that glutamine, tyrosine, and alanine were important in amino acids, and pyruvic‐ox‐2, ethylmalonic‐2, and phenyllactic‐2 were important in fatty acids. The area under the curve of body mass index standard deviation  score (BMI‐SDS) combined with key amino acid metabolites and fatty acid metabolites for predicting IR was 80.0% and 76.6%, respectively.

**Conclusions:**

There are common key metabolites related to IR and obese children, and these key metabolites combined with BMI‐SDS could effectively predict the risk of IR.

## Graphical abstract

Highlights

- Childhood obesity was significantly associated with the increased risk of insulin resistance (IR).
- There were 12 metabolites co‐associated with childhood obesity and IR, including seven amino acid and five fatty acid metabolites.
- The combination of body mass index standard deviation score and key metabolites could effectively predict the risk of IR.

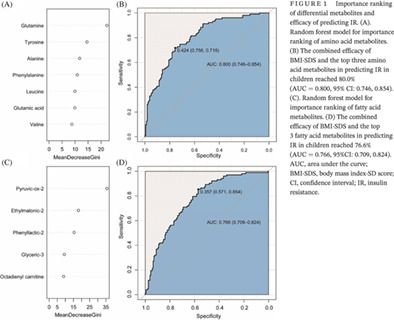

## 摘要

背景:儿童肥胖已成为严重的公共卫生挑战，胰岛素抵抗(IR)是其常见并发症之一。肥胖和IR都被认为是代谢紊乱的基础。然而，目前尚不清楚与儿童肥胖和IR相关的常见关键代谢物

方法:将儿童分为正常体重和超重/肥胖组。测量空腹血糖和空腹胰岛素，计算胰岛素抵抗稳态模型评估指数(HOMA‐IR)。应用液相色谱‐串联质谱进行代谢组学分析。通过多元线性回归分析和相关性分析探索肥胖、IR和代谢物之间的关系。使用随机森林(RF)对差异代谢物的重要性进行排序，并使用相对操作特征曲线进行预测

结果:共有88名正常体重儿童和171名肥胖/超重儿童参与了本研究。两组间有30种代谢物存在显著差异。儿童肥胖与10种氨基酸代谢物和20种脂肪酸代谢物显著相关。12种代谢物与IR显著相关。RF中代谢物的排名显示，谷氨酰胺、酪氨酸和丙氨酸在氨基酸中很重要，丙酮酸‐2、乙基丙二酸‐2和苯乳酸‐2在脂肪酸中很重要。BMI‐SDS曲线下面积与关键氨基酸代谢物和脂肪酸代谢物相结合，预测IR的准确率分别为80.0%和76.6%

结论:存在着与IR和肥胖儿童相关的关键代谢物，这些关键代谢物与BMI‐SDS一起，可以有效预测IR风险

## 1 INTRODUCTION

Childhood obesity is one of the largest public health problems around the world. The World Health Organization reported that the number of obese children in the world had increased more than 10‐fold in the last 40 years.^1^ Recently, China has become one of the countries that developed the fastest rising rate of childhood obesity. The report on nutrition and chronic diseases of Chinese residents (2020) showed that the proportion of overweight and obesity among children under 6 years old and adolescents between 6 and 17 years old had reached 19.0% and 10.4%, respectively.^2^ With the prevalence of childhood obesity, metabolic diseases tend to occur at a younger age and gradually spread to childhood. Childhood obesity not only affects children's physical health and quality of life, but also has adverse effects on health in adulthood, which could increase the risk of obesity, cardiovascular disease, diabetes, and even death.^3^ Among them, metabolic abnormalities are widely considered as serious issues that threaten the health of obese children and adolescents, and insulin resistance (IR) is closely related to metabolic abnormalities.^4^ In fact, IR may be an important cause of type 2 diabetes and cardiovascular disease among obese population so that it deserves to be paid more attention.

As one of the main characteristics of metabolic syndrome, incidence rate of IR is gradually increasing among children and adolescents, especially for obese population. It had been considered as an impaired biological response to insulin stimulation in target tissues, primarily liver, muscle, and adipose tissue. In addition, IR may impair glucose metabolism and then lead to a compensatory increase of insulin secretion and hyperinsulinemia in beta cells.^5^, ^6^, ^7^ Although genetic causes had also been identified, IR is primarily an acquired condition associated with excess body fat. Moreover, once insulin sensitivity decreased, the aggravation of IR and related complications would appear as results.

Metabolic change is one of key features of obesity as well as metabolic abnormalities is also closely related to IR, dyslipidemia, changes in glucose metabolism and type 2 diabetes mellitus.^8^, ^9^, ^10^ Insulin sensitivity changes constantly at different stages of children's growth and development. With the development of puberty and the change of sex hormone secretion, insulin sensitivity decreases significantly, accompanied by compensatory insulin secretion, and returns to normal after puberty.^11^ As a tool for the study of human disease, metabolomic approaches have increased the interest of clinicians and scholars in exploring possible biomarkers for cardiometabolic diseases.^12^, ^13^ Therefore, we explored the key metabolites associated with obesity and IR in children based on metabolomics.

In this study, we compared the incidence of IR in normal weight and obese children and screened for changes in key metabolites common to obesity and IR. Our study provided potential biomarkers for progression, diagnosis, and evaluation of the obesity combined with IR among children and is also of the great significance for the prevention of IR in obese children.

## 2 METHODS

### 2.1 Study population

Our study included children and adolescents aged between 6 and 14 years old as well as treated in the department of children's health care and endocrinology of the Children's Hospital of Nanjing Medical University. We excluded some objects of pathological obesity due to genetic diseases, metabolic diseases, and neuroendocrine diseases. The study had been approved by the Ethics Committee of Children's Hospital of Nanjing Medical University (201603004‐1). The purpose of study was explained to children's parents before thestart and informed consent was obtained as well.

### 2.2 Anthropometric measurement

Well‐trained medical staff had carried out all of physical measurements for children, including height, weight, waist circumference (WC), etc. Height and weight were measured by electronic post scale. Children were asked to be upright and wear only underwear. The height and weight were measured accurately to 0.1 cm and 0.1 kg. Body mass index (BMI) was calculated as weight (kilograms) divided by the square of height (meters), and BMI was converted to SD score of BMI (BMI‐SDS), as it corrects for variations in age and gender in children and allows for pooling of data. BMI‐SDS was grouped as follows: – 2 ≤ BMI‐SDS <1 indicates normal weight, 1 ≤ BMI‐SDS <2 indicates overweight, and BMI‐SDS ≥2 indicates obesity. In our study, the subjects were assigned to normal weight and overweight/obese groups. WC was measured to the nearest 0.1 cm at the level of the navel with a flexible inch tape while the subject was at the end of exhalation. The waist‐to‐height ratio was calculated as the WC (in centimeters) divided by height (in centimeters).

### 2.3 Laboratory examination

Venous blood (3 mL) was drawn from the participants in the morning after fasting for 12 h, the fasting blood glucose (FBG) was measured by hexokinase method, the fasting blood insulin (FBI) was measured by radioimmunoassay, and IR was calculated. Homeostasis model assessment of insulin resistance (HOMA‐IR) = FBI (mU/L) × FBG ([mmol/L]/22.5) was calculated. IR was considered with HOMA‐IR ≥3.0.^14^

Liquid chromatography–tandem mass spectrometry was used to analyze acylcarnitine on dried blood filter paper. First, venous blood was dripped onto a piece of filter paper that was about 10 mm in diameter. After drying at room temperature, the sample was stored for testing in refrigerator under −20°C. Then, 3 mm of dry blood filter paper was took and placed in a 96 perforated polypropylene plate. Then, 100 μL of internal standard methanol was added into each hole as well as placed at room temperature for 20 min. After that, acylcarnitine was extracted from blood filter paper and centrifuged in another 96 perforated polypropylene plate that was dried via nitrogen under 55°C. Then 60 μL of hydrochloric acid (3 mol/L) n‐butyl alcohol was added. By covering with Teflo film and placing in a 65°C incubator for 15 min, acylcarnitine was converted into acylcarnitine butyl ester, dried via nitrogen under 55°C, and then resuspended with 100 μL of 80% acetonitrile for testing. Quantitative analysis was performed by software (Chem View B5, Bio Application Systems, USA), which automatically calculated the concentration of acylcarnitine in the measured samples according to the ion peak and intensity of internal standard. The components of organic acids in urine had determined and analyzed by gas chromatography–mass spectrometry (GC–MS). GC–MS solution 2.72 data acquisition software was used for data processing and the semiquantitative results were obtained.

### 2.4 Statistical analysis

The KolmogorowSmironov tests were used to test the normality of the quantitative data. Those conforming to normal distribution were expressed as mean ± SD and comparison between groups used *t* test; The data of skewed distribution were represented by median (P_25_, P_75_), and compared by Mann–Whitney *U* test. Qualitative data were expressed as frequency (%), and chi‐square test was used for comparison. After the exclusion of metabolites with a detection rate <30%, square root transformation was performed on the data of metabolites to correct skewed distribution. Orthogonal partial least square discriminant analysis (OPLS‐DA) model was used to assess the difference in metabolites between the normal weight group and obese group. Nonparametric test and variable importance in project (VIP) were combined to screen the differential metabolites between the two groups.

Multiple linear regressions were used for exploring the relationships between childhood obesity and metabolites, with the obesity group as the independent variable and metabolites as dependent variables. Spearman correlation was used to analyze the associations between metabolites and IR among children. When the univariate correlation analysis coefficient was significant, the partial correlation analysis was performed after adjusting for age, gender, and BMI. Further, the random forest (RF) model was applied for predicting the importance of metabolites to childhood IR. RF model is an ensemble learning model that operates by building various decision trees during the training period and identifying the multiple classes depending on majority vote in the ensemble model. The common differential metabolites were considered as independent variables and the childhood IR was taken as dependent variables. The data set was adopted with 10‐fold cross‐validation. The RF model was implemented using the “random forest” package. The graphical relative operating characteristic (ROC) curve is produced, and the area under the ROC curve (AUC) is a performance index to measure the effectiveness of the model. A *p* value <.05 based on two‐tailed test results should be considered statistically significant. All analyses were performed with R. version 3.2.2.

## 3 RESULTS

Among the participants in the analysis, 88 of children with normal weight were 10.53 ± 2.41 years old, and 171 of children with obesity were 10.73 ± 1.98 years old. There was no significant difference in age and gender distribution between two groups. In addition, BMI, (WC, waist‐to‐height ratio, and hip circumference were significantly higher in obese children than those in the control group. Impaired fasting blood glucose showed no difference between two groups, but IR did (Table 1).

**TABLE 1:** Children's characteristics and physical measures.

	Control (n = 88)	Obesity (n = 171)	*t*/*χ* ^*2*^/z	*p* values
Age (years)	10.53 ± 2.41	10.73 ± 1.98	−0.657	.512
Gender				
Male	58 (65.9)	115 (63.7)	0.047	.828
Female	30 (34.1)	56 (32.7)		
BMI	15.67 (14.19, 18.06)	27.70 (25.70, 30.40)	−13.112	**<.001**
BMI‐SDS	−0.62 ± 1.15	3.08 ± 0.62	−28.194	**<.001**
WHtR	0.42 (0.40, 0.45)	0.60 (0.56, 0.64)	−12.757	**<.001**
IFG				
No	84 (95.5)	166 (97.1)	0.455	.751
Yes	4 (4.5)	5 (2.9)		
IR				
No	78 (88.6)	78 (45.6)	44.896	**<.001**
Yes	10 (11.4)	93 (54.4)		

*Note*: *p* values < .05 are bolded.

Abbreviations: BMI‐SDS, body mass index‐SD score; IFG, impaired fasting glucose; IR, insulin resistance; WC, waist circumference; WHtR, waist‐to‐height ratio.

The chromatogram showed stable retention time with good reproducibility, which means the metabolomic analysis had outstanding reliability. A total of 195 metabolites were detected, and 111 of metabolites were included in the analysis, with a detection rate higher than 30%. OPLS‐DA made the results be more intuitive. The results showed that there was no crossover of metabolites between normal and obese group, which could be well distinguished and had statistical significance (R^2^ = 0.864, Q^2^ = 0.824) (Figure S1).

Combined with VIP values, Mann–Whitney *U* test was determined markers of difference between two groups. VIP value was identified the relative contribution of each metabolite in order to distinguish different groups. In addition, the VIP value was proportional to its contribution, and the threshold was set as VIP >1. Nevertheless, *p* values of the Mann–Whitney *U* test had reflected statistical differences in the level of metabolic small molecules. The results showed that there were significant differences in 30 metabolites between the normal weight group and obese group (Table S1).

Table 2 showed that children's obesity was associated with 30 types of metabolites. Among the amino acid metabolites, childhood obesity was significantly negatively correlated with glutamine (*β* = −1.675, 95% confidence interval [CI]: −1.936, −1.414), citrulline (*β* = −0.415, 95% CI: −0.545, −0.284), and argine (*β* = −1.210, 95% CI: −1.510, −0.910). In terms of fatty acid metabolites, childhood obesity increased the concentration of octadecadienyl carnitine (*β* = 0.048, 95% CI: 0.029, 0.066), oleylcarnitine (*β* = 0.099, 95% CI: 0.069, 0.128), and palmitic‐1 (*β* = 1.472, 95% CI: 1.081, 1.863), etc.

**TABLE 2:** Associations between obesity and metabolites in children.

Metabolites	*β* (95% CI)	*p* value
Amino acid		
Phenylalanine	0.744 (0.505, 0.983)	**<.001**
Alanine	1.706 (1.215, 2.196)	**<.001**
Glutamic acid	1.147 (0.684, 1.610)	**<.001**
Glutamine	−1.675 (−1.936, −1.414)	**<.001**
Citrulline	−0.415 (−0.545, −0.284)	**<.001**
Argine	−1.210 (−1.510, −0.910)	**<.001**
Tyrosine	0.951 (0.743, 1.160)	**<.001**
Leucine	1.016 (0.661, 1.371)	**<.001**
Valine	1.008 (0.698, 1.319)	**<.001**
Histidine	−3.828 (−4.420, −3.236)	**<.001**
Fatty acid		
Octadecadienyl carnitine	0.048 (0.029, 0.066)	**<.001**
3‐Hydroxyhexyl carnitine	0.028 (0.020, 0.037)	**<.001**
2‐Hexenedioic‐2	−0.646 (−0.826, −0.467)	**<.001**
5‐Oxoproline‐2	−0.420 (−0.548, −0.292)	**<.001**
Phenyllactic‐2	0.809 (0.717, 0.900)	**<.001**
Pyruvic‐ox‐2	1.241 (0.912, 1.570)	**<.001**
Oxalic‐2	−0.403 (−0.505, −0.300)	**<.001**
Glyceric‐3	0.744 (0.528, 0.959)	**<.001**
Succinic‐2	−0.717 (−0.936, −0.498)	**<.001**
Hippuric‐2	−3.444 (−3.902, −2.986)	**<.001**
Ethylmalonic‐2	0.130 (0.040, 0.220)	**.005**
Glyoxylic‐ox‐2	−0.864 (−1.053, −0.675)	**<.001**
Palmitic‐1	1.472 (1.081, 1.863)	**<.001**
Propionyl carnitine	0.125 (0.056, 0.193)	**<.001**
Octadienyl carnitine	0.031 (0.020, 0.043)	**<.001**
Oleylcarnitine	0.099 (0.069, 0.128)	**<.001**
Octenyl carnitine	0.076 (0.050, 0.103)	**<.001**
Palmitoleyl carnitine	0.045 (0.033, 0.057)	**<.001**
Palmitoyl carnitine	0.076 (0.046, 0.106)	**<.001**
N‐Acetylaspartic‐2	0.145 (0.081, 0.210)	**<.001**

*Note*: *p* values < .05 are bolded.

Abbreviation: CI, confidence interval.

Furthermore, Spearman correlation and partial analyses explored the correlation between 30 types of metabolites and IR. Coefficient 1 was the result of univariate correlation analysis, and coefficient 2 adjusted potential variables. The results showed that 12 metabolites had significant correlation with IR, including seven amino acid metabolites and five fatty acid metabolites (Table 3).

**TABLE 3:** Correlation between metabolites and IR in children.

Metabolites	Coefficient 1^a^	*p* value	Coefficient 2^b^	*p* value
Amino acid				
Phenylalanine	0.352	**<.001**	0.224	**<.001**
Alanine	0.395	**<.001**	0.222	**<.001**
Glutamic acid	0.206	**.001**	0.192	**.002**
Glutamine	−0.397	**<.001**	−0.286	**<.001**
Citrulline	−0.222	**<.001**	−0.091	.146
Argine	−0.280	**<.001**	−0.093	.136
Tyrosine	0.366	**<.001**	0.199	**<.001**
Leucine	0.386	**<.001**	0.208	**.001**
Valine	0.345	**<.001**	0.165	**.008**
Histidine	−0.230	**<.001**	−0.044	.481
Fatty acid				
Octadienyl carnitine	0.229	**<.001**	0.128	**.041**
3‐Hydroxyhexyl carnitine	0.179	**.004**	0.042	.500
2‐Hexenedioic‐2	−0.172	**.016**	−0.005	.937
5‐Oxoproline‐2	−0.170	**.016**	−0.027	.665
Phenyllactic‐2	0.395	**<.001**	0.227	**<.001**
Pyruvic‐ox‐2	0.413	**<.001**	0.192	**.002**
Oxalic‐2	−0.161	**.009**	0.019	.760
Glyceric‐3	0.236	**<.001**	0.191	**.002**
Succinic‐2	−0.190	**.002**	0.037	.560
Hippuric‐2	−0.308	**<.001**	−0.026	.677
Ethylmalonic‐2	0.189	**.002**	0.163	**.009**
Glyoxylic‐ox‐2	−0.222	**<.001**	−0.036	.563
Palmitic‐1	0.273	**<.001**	0.100	.110
Propionyl carnitine	−0.047	.453	/	/
Octadecadienyl carnitine	0.053	.393	/	/
Oleylcarnitine	0.071	.255	/	/
Octenyl carnitine	0.095	.128	/	/
Palmitoleyl carnitine	0.055	.380	/	/
Palmitoyl carnitine	0.063	.310	/	/
N‐Acetylaspartic‐2	0.025	.690	/	/

*Note*: *p* values < .05 are bolded.

Abbreviations: BMI, body mass index; CI, confidence interval.

^a^ Univariate correlation.

^b^ Partial correlation, adjusted for child's age, gender, and BMI.

**FIGURE 1:**
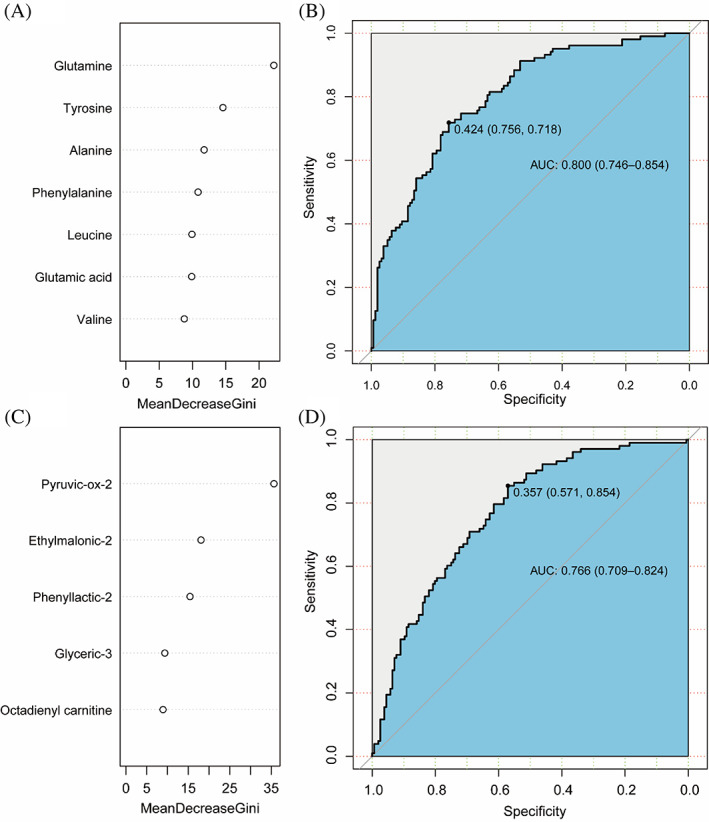
Importance ranking of differential metabolites and efficacy of predicting IR. (A). Random forest model for importance ranking of amino acid metabolites. (B) The combined efficacy of BMI‐SDS and the top three amino acid metabolites in predicting IR in children reached 80.0% (AUC = 0.800, 95% CI: 0.746, 0.854). (C). Random forest model for importance ranking of fatty acid metabolites. (D) The combined efficacy of BMI‐SDS and the top 3 fatty acid metabolites in predicting IR in children reached 76.6% (AUC = 0.766, 95%CI: 0.709, 0.824).AUC, area under the curve; BMI‐SDS, body mass index‐SD score; CI, confidence interval; IR, insulin resistance.

A total of differential metabolites co‐associated with childhood obesity and IR were divided into amino acid metabolites and fatty acid metabolites. The RF models were used to explore the effects of amino acid metabolites (Figure 1A) and fatty acid metabolites (Figure 1C) on IR, and their importance was ranked. The combined efficacy of BMI‐SDS and the top three amino acid metabolites including glutamine tyrosine and alanine in predicting IR in children reached 80.0% (95% CI: 0.746, 0.854) (Figure 1B). In terms of fatty acid metabolites, The AUC of BMI‐SDS combined with pyruvic‐ox‐2, ethylmalonic‐2, phenyllactic‐2 was 76.6% (95% CI: 0.709, 0.824). (Figure 1D). Finally, the combined efficacy of BMI‐SDS with the top three amino acid metabolites and the top three fatty acid metabolites in predicting IR reached 80.4% (AUC = 0.804, 95% CI: 0.750, 0.857) in children (Figure S2).

## 4 DISCUSSION

This study showed that there were significant differences in 30 types of metabolites between obese and normal children, 12 of which also associated with IR. In fact, the key metabolites were associated with increased risk of IR among obese children. Based on the importance of key metabolites for IR, we ranked the importance of metabolites and evaluated their performance in predicting IR, and confirmed the outstanding predictive efficacy.

Human metabolism had a close relationship with obesity. A recent meta‐analysis of obesity and metabonomic had shown that the level of leucine, isoleucine, valine, tyrosine, phenylalanine, glutamate, lysine, and alanine increased among obese individuals, whereas glycine levels decreased. This is similar to our results, indicating that there were significant differences in amino acid levels between obese and normal populations.^15^ In obese subjects, the increase of some amino acid levels may be related to the low expression of L‐type amino acid transporter 1 protein, which transports large neutral amino acids such as ranched‐chain amino acids, phenylalanine, and tyrosine.^16^ Regarding changes in the number of aromatic amino acids including phenylalanine and tyrosine in obese individuals, it was proposed that the metabolic disorder caused by obesity would lead to liver dysfunction, accompanied by the reduction of phenylalanine and tyrosine metabolism and ultimately increase their levels in the blood.^15^ Glutamic acid is a basic substance for energy metabolism associated with metabolic diseases. The high levels of glutamate in obese individuals are due to lower absorption of the tricarboxylic acid cycle. In addition, glutamic acid may also be triggered by glucagon release from alpha‐pancreatic cells, aggravating metabolic diseases.^17^ As a kind of ester compound synthesized by carnitine and fatty acid, acylcarnitine can reflect the metabolism of fatty acid. We also found that obese children had higher concentrations of pyruvic‐ox‐2 and glyceric‐3. As an important intermediate in the metabolism of sugar, lipid, and amino acid, pyruvate is mainly derived from carbohydrates. It is also an accelerator of gluconeogenesis and increases the content of glucose in the blood.^18^ In addition, as one of the common organic acids, glyceric acid can be used in cooking of bread and other foods, children who are overweight or obese tend to consume high‐calorie foods, so the glyceric acid derivative phosphoglyceric acid is enhanced in the body.

IR is the basis of metabolic disorders and complications caused by obesity. Childhood IR is more likely to accelerate the occurrence and development of nonalcoholic fatty liver disease, type 2 diabetes, and cardiovascular disease, and their potential mechanism involves complex metabolic pathways.^10^, ^19^, ^20^ A cohort study indicated that significant changes in branched‐chain amino acids, phenylalanine, alanine tyrosine, and tryptophan among children with IR.^21^ In terms of the relationship between key amino acids and IR, it had been reported that the levels of elevated glutamate are positively associated with BMI, childhood obesity,^22^ and IR,^23^ whereas glutamine was lower among children with obesity.^24^ This is consistent with our study as well. Because of changing in gut microbiota environment of obese children, metabolism of glutamate and glutamine in the body might be affected so that lead to changes in plasma amino acids. Gluconeogenesis would cause increased hepatic glucose output as well as causes IR. Childhood obesity and IR individual participant based meta‐analysis showed that a sphingomyelin was positively associated with obesity, whereas increased alanine and tyrosine had been correlated with HOMA, suggesting their roles in gluconeogenesis and insulin resistance. Tyrosine is biosynthesized from the indispensable amino acid phenylalanine, and elevated tyrosine concentrations may be due to elevated tyrosine transaminase activity caused by increased insulin secretion.^25^ Studies showed that leucine, glutamine, and alanine are responsible for amino acid hypersensitivity in islets^26^; amino acid oxidation via glutamate dehydrogenase produces ATP and triggers insulin secretion. The signaling effect of amino acids amplifies insulin release after *β*‐cell depolarization and elevation of cytosolic calcium.^27^ Inhibition of glutamate dehydrogenase activity is involved in regulating insulin secretion in specific fatty acid oxidation disorders.^26^

In addition, pyruvate is a precursor for gluconeogenesis and the biosynthesis of glycerol, fatty acids, and nonessential amino acids. The elevated pyruvate level suggests a deficiency of pyruvate dehydrogenase, which is required for acetyl‐CoA production.^28^ It has also been observed that increased lactate reflects dysregulation of central carbon metabolism and that obesity is closely associated with adipocyte hypertrophy, which is associated with local hypoxia promoting lactate production.^29^ Thus, increased pyruvate and lactate levels may form characteristic markers of obesity and IR in children.^28^ Existing evidence has shown that high‐fat diet promoted fructose metabolism and increased the levels of glyceric acid, damaging the secretion and function of islet *β*‐cells and accelerating the occurrence of type 2 diabetes.^30^

Notably, adolescent development is one of the important physiological factors affecting IR. At this stage, the increase of growth hormone and sex hormone levels is accompanied by a transient decrease of insulin sensitivity and compensatory increase of insulin secretion. Therefore, after adjusting for age, gender, and BMI, we identified common metabolites associated with childhood obesity and IR and predicted IR based on key common metabolites, including glutamine, tyrosine, alanine, pyruvic‐ox‐2, ethylmalonic‐2, and phenyllactic‐2, which likely have the potential to be early markers of the onset of IR. Ethylmalonic‐2 is a major and potentially cytotoxic metabolite associated with short‐chain acyl‐CoA dehydrogenase deficiency,^31^ yet its role of the relationship between obesity and IR is still unclear. Our study provides new ideas for exploring the relationships between ethylmalonic‐2 and IR, and further research is needed to verify this in the future.

Major strengths of this study include that we compared the differences in IR between obese and normal weight children by taking into account age, and we explored the key metabolites associated with childhood obesity and IR, which may have implications for the prevention of childhood obesity and complications. Second, we jointly predicted IR based on the results of RF machine learning, which provides promising prospects for the diagnosis and treatment of IR. Indeed, our findings highlighted the needs for comprehensive consideration variables in studies of IR children as well as more mechanism experiments to approve the results of epidemiological studies.

There were some limitations in our study. As a cross‐sectional study, the causal relationships between metabolites and IR cannot be established in this study. We cannot ignore other possible confounders that were associated with IR, and it requires further confirmation in following longitudinal studies. In addition, the number of control group is less than that of obese children in this study. However, it had shown that the comparison results between the two groups were still stable. Future studies needed to further verify the potential mechanisms of the key metabolites and the occurrence of IR, as well as how to coordinate and modify the levels of these metabolites in the body.

## 5 CONCLUSION

In summary, the risk of IR significantly increased among obese children. Multiple linear regression models and correlation analyses were applied for assessing the associations between key metabolites and obesity as well as IR. By giving the results of these two models, we concluded that key metabolites were associated with IR among obese children, just like the combination of key metabolites, including three amino acid metabolites (glutamine, tyrosine, alanine) and three fatty acid metabolites (pyruvic‐ox‐2, ethylmalonic‐2, phenyllactic‐2), have good efficacy in predicting IR. This study provides a reference for predicting IR among children.

## FUNDING INFORMATION

This work was supported by Jiangsu Provincial key research and development program (BE2015607), China Postdoctoral Science Foundation (2022 M721683).

## CONFLICT OF INTEREST STATEMENT

The authors declare that they have no known competing financial interests or personal relationships that could have appeared to influence the work reported in this paper.

## Supporting information


**Data S1:** Supporting Information.

## Data Availability

The data that support the findings of this study are available on request from the corresponding author. The data are not publicly available due to privacy or ethical restrictions.
