# Supplementary material for: The link between obesity and insulin resistance among children: Effects of key metabolites
Source: J Diabetes. 2023 Aug 25;15(12):1020–8. doi: 10.1111/1753-0407.13460 (PMC10755598; doi:10.1111/1753-0407.13460)
Supplement: Supplementary file 1 — Data S1: Supporting Information. [file JDB-15-1020-s001.docx]

**The link between obesity and insulin resistance among children: Effects of key metabolites**

Wu Yan PhD^1#^, Su Wu PhD ^2#^, Qianqi Liu PhD ^1^, Qingqing Zheng MD^1^, Wei Gu PhD ^2^，Xiaonan Li PhD ^1,3^ *

^1^ Department of Children Health Care, Children's Hospital of Nanjing Medical University, Nanjing, 210008, China. yanwu@njmu.edu.cn (Wu Yan); 18951769617@163.com (Qianqi Liu); zqq102177@163.com (Qingqing Zheng); xiaonan6189@163.com (Xiaonan Li)

^2^ Department of endocrinology, Children's Hospital of Nanjing Medical University, Nanjing, 210008, China. wsuew@126.com (Su Wu); guwei154@njmu.edu.cn (Wei Gu)

^3^ Institute of Pediatric Research, Nanjing Medical University, Nanjing, 210029, China.

^#^ These authors have contributed equally to this work and share first authorship (Wu Yan, Su Wu).

**Corresponding author:**

Dr. Xiaonan Li

E-mail: xiaonan6189@163.com

Phone: +86-25-83117285


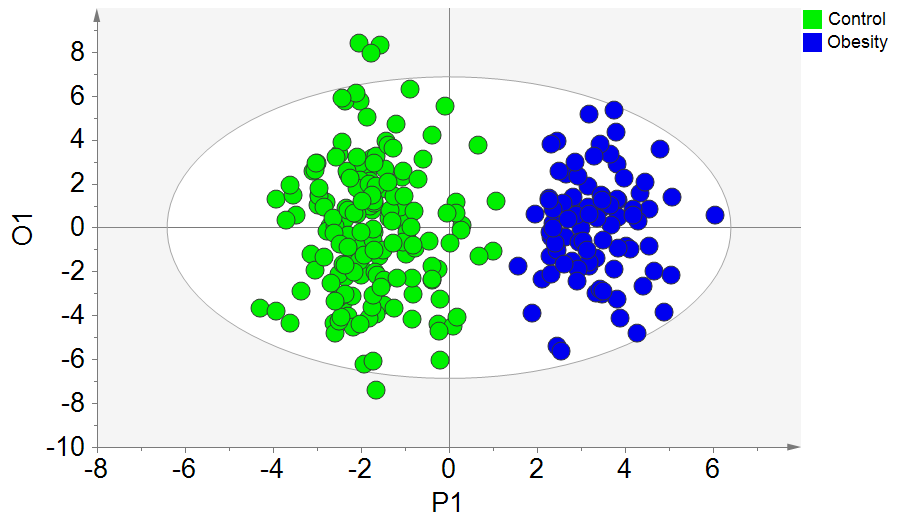


**Figure S1** Score plot of OPLS-DA. There was no crossover of metabolites between normal and obese group, which could be well distinguished and had statistical significance


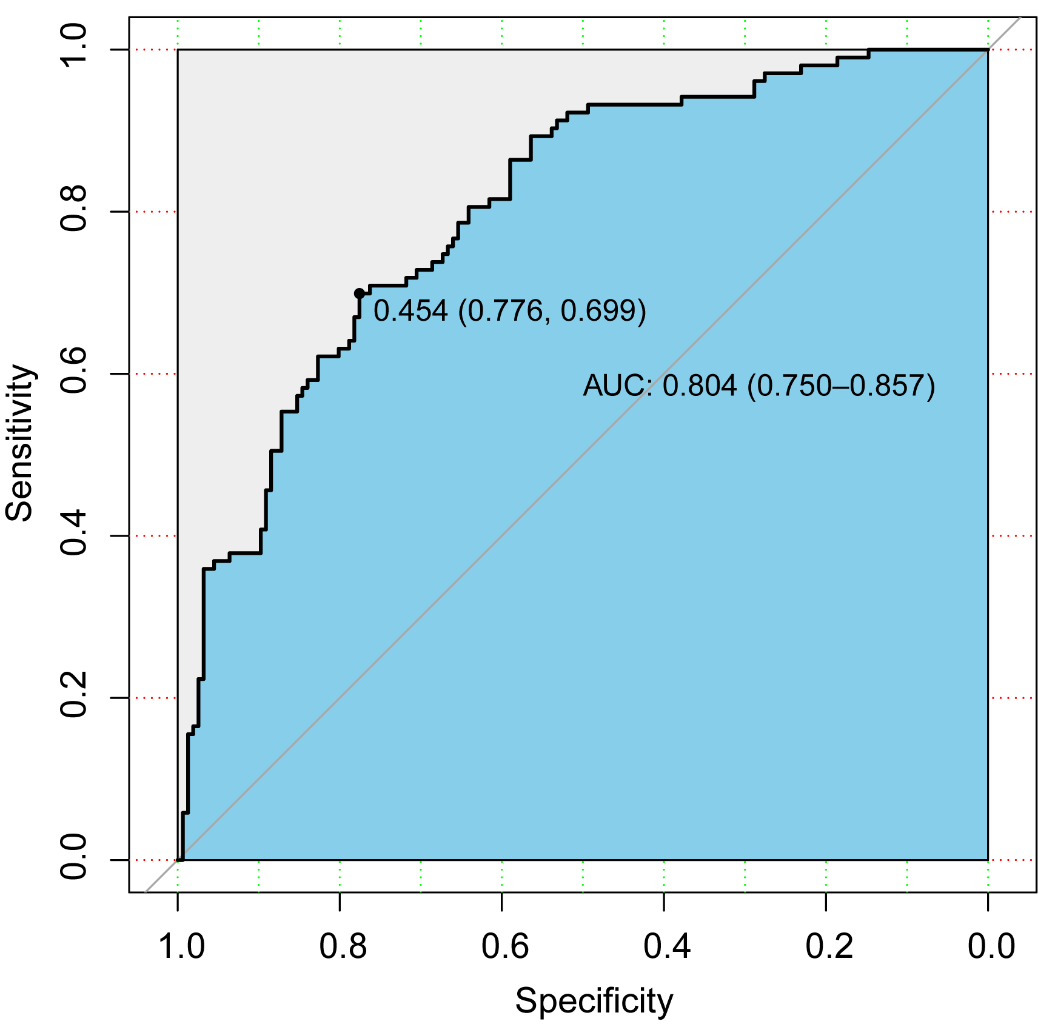


**Figure S2** The combined efficacy of BMI-SDS, the top 3 fatty acid metabolites, and the top 3 fatty acid metabolites in predicting IR in children reached 80.4% (AUC=0.804, 95%CI: 0.750, 0.857).

**Table S1 Screening for differential metabolites in obese and control children**

| Metabolites | Mann-Whitney *U* test  *P-values* | OPLS-DA  VIP＞1 |
| --- | --- | --- |
| Phenyllactic-2 | **＜0.001** | **2.962** |
| Glutamine | **＜0.001** | **2.920** |
| Histidine | **＜0.001** | **2.782** |
| Hippuric-2 | **＜0.001** | **2.367** |
| Glyoxylic-OX-2 | **＜0.001** | **2.182** |
| Tyrosine | **＜0.001** | **2.085** |
| Argine | **＜0.001** | **1.872** |
| Palmitoleylcarnitine | **＜0.001** | **1.825** |
| Palmitic-1 | **＜0.001** | **1.751** |
| Alanine | **＜0.001** | **1.701** |
| Oleylcarnitine | **＜0.001** | **1.657** |
| 3-Hydroxyhexyl carnitine | **＜0.001** | **1.628** |
| Valine | **＜0.001** | **1.587** |
| Citrulline | **＜0.001** | **1.550** |
| Oxalic-2 | **＜0.001** | **1.547** |
| Succinic-2 | **＜0.001** | **1.524** |
| Phenylalanine | **＜0.001** | **1.520** |
| 5-Oxoproline-2 | **＜0.001** | **1.511** |
| Leucine | **＜0.001** | **1.412** |
| Glyceric-3 | **＜0.001** | **1.405** |
| Glutamic acid | **＜0.001** | **1.324** |
| Palmitoylcarnitine | **＜0.001** | **1.321** |
| Octadecadienyl carnitine | **＜0.001** | **1.304** |
| 2-Hexenedioic-2 | **＜0.001** | **1.213** |
| Pyruvic-OX-2 | **＜0.001** | **1.170** |
| Octadienyl carnitine | **＜0.001** | **1.112** |
| Propionyl carnitine | **＜0.001** | **1.087** |
| N-Acetylaspartic-2 | **＜0.001** | **1.059** |
| Octenyl carnitine | **＜0.001** | **1.020** |
| Ethylmalonic-2 | **0.001** | **1.003** |

OPLS-DA: Orthogonal partial least square discriminant analysis; VIP: variable importance in project
